# Microbial Metabolite, Macro Impact: Urolithin A in the Nexus of Insulin Resistance and Colorectal Tumorigenesis

**DOI:** 10.3390/nu17233712

**Published:** 2025-11-26

**Authors:** Vennila Joseph, Slavomir Hornak, Peter Kubatka, Dietrich Büsselberg

**Affiliations:** 1 Weill Cornell Medicine-Qatar, Education City, Qatar Foundation, Doha Metropolitan Area, Doha P.O. Box 22104, Qatar; vsj4001@qatar-med.cornell.edu; 2 Center of Experimental and Clinical Regenerative Medicine, Small Animal Clinic, University of Veterinary Medicine and Pharmacy, 041 81 Kosice, Slovakia; slavomir.hornak@uvlf.sk (S.H.); peter.kubatka@uvlf.sk (P.K.)

**Keywords:** Urolithin A, colorectal cancer, type 2 diabetes, mitophagy, autophagy, apoptosis

## Abstract

Urolithin A (UA), a metabolite of dietary ellagitannins produced by the gut microbiome, is a potential dual-purpose bioactive compound that may interfere with the shared pathogenic pathways linking colorectal cancer (CRC) and type 2 diabetes mellitus (T2DM). This review summarizes recent preclinical and clinical data on UA’s mechanisms, therapeutic potential, and translational challenges. In CRC models, UA promotes G2/M cell cycle arrest, triggers both intrinsic and extrinsic caspase-mediated apoptosis, enhances CD8+ T-cell mitophagy and memory functions, suppresses Wnt/β-catenin signaling, and reduces chemoresistance, especially to 5-FU. For T2DM, UA enhances autophagic flux, mitophagy, insulin signaling, and GLUT4-mediated glucose uptake through the AMPK and PI3K/AKT pathways, reduces fasting glucose and insulin resistance in animal studies, and promotes adipose tissue browning and mitochondrial beta-oxidation. Human biomarker research is limited but indicates positive changes following interventions that increase UA. Future priorities include biomarker-driven, dose-finding trials stratified by metabotype, developing colon-targeted vs. systemic formulations, and testing combinations with chemotherapy and immunotherapy to determine safety and effectiveness.

## 1. Introduction

One major global health concern is the increasing prevalence of colorectal cancer (CRC) and type 2 diabetes (T2DM). Globally, over 589 million adults have T2DM, and this figure is expected to increase. T2DM has a significant economic impact, as evidenced by the 3.4 million deaths and the US $1 trillion financial burden it caused in 2024 [1]. With over 150,000 new cases and 52,900 deaths estimated anticipated in the USA in 2025 alone, CRC is currently the third most common cancer and the second largest cause of cancer-related death globally [2]. The high cost of CRC-related healthcare, estimated to exceed $24 billion annually in countries such as the United States, places a significant burden on patients and healthcare systems [3]. Beyond their rising incidence, the co-occurrence of T2DM and CRC in the same patients compounds morbidity, mortality, and health-economic burden [3].

According to a significant amount of epidemiological research, patients with T2DM face a 20–40% higher risk of developing CRC [4]. Shared risk factors and complex pathophysiological mechanisms are believed to drive this association. One such mechanism is hyperinsulinemia, a hallmark of insulin resistance in T2DM. It promotes cell division and inhibits apoptosis through the insulin-like growth factor 1 (IGF-1) signaling axis—a pathway often dysregulated in CRC [5]. Low-grade inflammation and gut microbial changes further enhance PI3K/AKT/mTOR and Wnt/β-catenin signaling, creating a tumor-promoting environment in insulin-resistant states. Given these interconnected molecular pathways, there is growing interest in nutritional pharmacology. Bioactive dietary substances that can influence human physiology to treat chronic diseases have gained more research interest. Polyphenols, a broad class of secondary metabolites found in plants, are commonly present in fruits, vegetables, and nuts, are among the promising candidates [6]. Ellagitannins (ETs) and their precursor ellagic acid (EA), found in pomegranates, berries, walnuts, and pecans, are poorly absorbed in the upper gut but are converted by colonic microbes into urolithins—highly bioavailable, active metabolites. This conversion is crucial for their bioactive effects [7]. Lin et al. (2023) demonstrated that only gut microbiota-derived UA is beneficial, not EA itself [8]. This places the gut microbiome, rather than the parent polyphenol, at the center of causal biology and translational variability.

Urolithins are dibenzo-α-pyrone derivatives. Urolithin A (UA) is one of the most studied urolithins and exists in several isoforms [9]. UA production varies across individuals because microbial conversion requires specific bacterial taxa (for example, Bifidobacterium) in the colon [10]. People can be classified as either producers (metabotype A or B, depending on the final urolithins produced) or non-producers (metabotype 0). This has led to the concept of “urolithin metabotypes.” This classification has important implications for clinical outcomes and personalized nutrition [11]. After formation, urolithins are absorbed and extensively metabolized in the liver via glucuronidation and sulfation. These conjugated forms—mainly glucuronides and sulfates—are the major circulating metabolites. Urolithin glucuronides are the dominant metabolites in urine and plasma [12]. Studies have shown that Neutrophil β-glucuronidase can cleave them, releasing aglycones (the unconjugated bioactive form of UA). The aglycone form can act on distant tissues such as the liver, muscle, pancreas, and colonic epithelium [13]. This “conjugate-to-aglycone” shuttle suggests tissue- and context-dependent regeneration of free UA at inflammatory or tumor sites, directly relevant to CRC and metabolic inflammation.

Urolithins, especially Urolithin A (UA), show consistent bioactivity across models, combining anti-inflammatory, antioxidant, antiproliferative, and pro-apoptotic actions. Mechanistically, UA targets key signaling pathways altered in cancer and metabolic disease, including PI3K/AKT/mTOR and AMPK [14,15]. In CRC models, UA induces G2/M arrest and caspase-dependent apoptosis, suppresses Wnt/β-catenin programs, dampens glycolytic flux via p53/TIGAR, and enhances 5-fluorouracil (FU) efficacy while modulating drug transporters [16,17,18,19,20,21]. In metabolic settings, UA activates AMPK, improves GLUT4 trafficking and insulin action, promotes adipose thermogenesis, and restores organelle quality via mitophagy/autophagy, a constellation aligned with improved insulin sensitivity [15,22,23,24]. UA’s ability to influence pathways relevant to both CRC and T2DM makes it a promising dual-purpose agent. However, translation to humans remains limited. Many questions remain, particularly regarding human clinical data, the optimal dosage, and the long-term safety of UA. Two translational variables are especially critical: (i) inter-individual metabotypes that govern endogenous UA generation, and (ii) extensive phase-II conjugation that limits free-UA exposure at target tissues [11,25]. These factors argue for biomarker-integrated clinical trials that stratify participants by metabotype and insulin-resistance status, and for the development of targeted delivery strategies (for example, colon-targeted versus systemic delivery) that balance anti-tumor and metabolic goals. This review critically synthesizes how UA affects (i) CRC (G2/M arrest, caspase-dependent apoptosis, suppression of Wnt/β-catenin and PI3K/AKT/mTOR, mitigation of chemoresistance) and (ii) T2DM (AMPK and PI3K/AKT activation, improved GLUT4 trafficking, mitophagy/autophagy, adipose thermogenesis, anti-inflammatory/antioxidant effects). It then maps shared CRC–T2DM nodes (insulin/IGF signaling, AMPK–mTOR crosstalk, inflammation, microbiome-driven biotransformation) to justify urolithins as dual-purpose modulators. Finally, the review discusses translational priorities and proposes biomarker-guided trials with colon-targeting drug delivery systems. In short, we position UA as a microbiome-derived molecule with bidirectional actions on tumor biology and insulin resistance, warranting rigorous clinical interrogation.

## 2. Materials and Methods

Protocol and reporting. We conducted a narrative review to evaluate effect of UA in two disease domains: CRC and T2DM.

Information sources and search date. PubMed and Scopus were searched from database inception to 7 July 2025.

Search strategy. We combined controlled keywords for the exposure and disease domains using Boolean operators. The operational query included the terms: “urolithin A” AND (“insulin resistance” OR “insulin sensitivity” OR “HOMA-IR” OR “glucose tolerance” OR “insulin signaling” OR “metabolic syndrome” OR “type 2 diabetes” OR “glucose”) and “urolithin A” AND (“colorectal cancer” OR “colon cancer” OR “CRC”). Searches were run separately for the T2DM and CRC domains and then cross-queried for overlap. PubMed returned 40 UA–T2DM and 27 UA–CRC records; Scopus returned 53 UA–T2DM and 38 UA–CRC records; the combined UA–CRC–T2DM query identified 3 records in Scopus and 0 in PubMed.

Eligibility criteria. We included primary research articles in English that evaluated UA (any form/exposure) in CRC and/or T2DM/metabolic contexts and reported mechanistic and/or phenotypic outcomes (e.g., cell-cycle/apoptosis, Wnt/β-catenin or PI3K/AKT/mTOR signaling; glucose uptake/insulin signaling, AMPK activation, mitophagy/autophagy). We excluded systematic reviews and non-English articles on these topics.

Study selection. The initial combined yield was 161 records. After excluding 4 non-English records and removing duplicates, 97 records remained for full-text assessment. Of these, 36 were reviews and were excluded, leaving 61 primary studies for qualitative synthesis. Titles/abstracts were screened, followed by full-text evaluation against the criteria above.

Data extraction and items. From each eligible study, we extracted: authors/year; model/system (in vitro, in vivo, clinical/biomarker); disease context (CRC vs. T2DM); UA form/dose/exposure; primary readouts (mechanistic pathways and functional outcomes); and key findings.

Synthesis approach. Given heterogeneous models, exposures, and endpoints, we performed a narrative synthesis with thematic grouping by disease domain (CRC vs. T2DM) and by mechanistic axes (e.g., Wnt/β-catenin, PI3K/AKT/mTOR, AMPK, mitophagy/autophagy, inflammatory/oxidative pathways). No quantitative meta-analysis was attempted.

Outcome of interest. The primary outcomes were direction and consistency of UA effects on (i) tumor-relevant hallmarks in CRC and (ii) insulin action and metabolic homeostasis in T2DM, with attention to dose/exposure, model dependency, and mechanistic plausibility.

## 3. Results

### 3.1. Urolithin A and Colorectal Cancer

#### 3.1.1. Cell Viability and Apoptosis

UA primarily inhibits CRC by inducing apoptosis in cancer cells. This has been demonstrated across several human CRC cell lines, including HCT116 (p53 wild-type), SW620 (p53-mutant metastatic), HT-29 (p53/BRAF-mutant), and Caco-2 (p53-mutant). The antiproliferative effect of UA is dose- and time-dependent. For example, the IC_50_ in HCT116 cells decreases from 39.2 µM at 48 h to 19.6 µM at 72 h, indicating increased efficacy over time [18]. The ability of UA to inhibit growth in various cancer cell lines was also found to depend on its conjugation state. HT-29 cells exhibit relative resistance due to rapid glucuronidation, whereas HCT116 cells retain higher intracellular levels of active UA due to slower metabolism [8,19]. A study by Gonzalez-Sarrias et al. (2017) showed that UA was more effective than its isomer, Isourolithin A, in Caco-2 cells, with an IC_50_ of 49.2 µM after 48 h [26]. This difference was linked to faster glucuronidation of isourolithin A, whose conjugated forms show much lower antiproliferative activity than the unconjugated compound [19,25]. Both UA and its isomer showed some selectivity for cancer cells, as they are considerably less damaging to normal human colon fibroblasts than to CRC cells [16,26].

Complementing these in vitro findings, Tortora et al. (2018) provided in vivo evidence that dietary supplementation with pomegranate mesocarp decoction, a rich source of UA precursors, reduced preneoplastic mucin-depleted foci in Apc-mutated Pirc rats [27].

**Figure 1:**
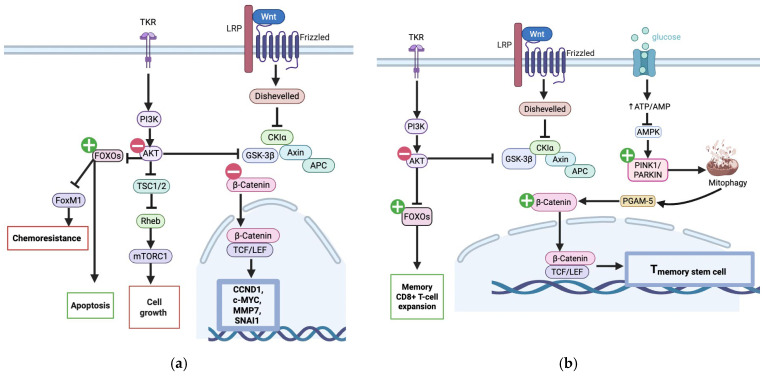
Pathways modulated by UA in CRC: (**a**) In CRC cells, UA inhibits PI3K/AKT/mTOR and Wnt/β-catenin signaling, promoting apoptosis and reducing chemoresistance, proliferation, and metastasis. (Created in BioRender. Busselberg, D. (2025) https://BioRender.com/8z6f48a, accessed on 08 November 2025) (**b**) In T cells, UA enhances memory CD8+ T-cell expansion via PI3K/AKT/mTOR and Wnt/β-catenin pathway via mitophagy-associated signaling. (Created in BioRender. Busselberg, D. (2025) https://BioRender.com/ld15v1o accessed on 8 November 2025). 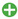: UA mediated stimulation, 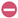 UA mediated inhibition, **┴**: inhibition. c.

UA induces G2/M phase cell-cycle arrest by increasing p53 and its downstream effector p21, a cyclin-dependent kinase inhibitor [16,28]. Studies using p53-knockout HCT116 cells, in which the growth-inhibitory effects of UA were markedly reduced, illustrate the crucial role of UA in this p53 pathway [18]. Additionally, UA’s ability to suppress the PI3K/Akt/mTOR pathway contributes significantly to its antiproliferative effects in CRC. Tiwari et al. (2024) demonstrated, using network pharmacology and molecular docking, that UA binds to and inhibits the PI3K/AKT/mTOR pathway, leading to downstream suppression of oncogenic signaling (Figure 1a) [28].

**Figure 2:**
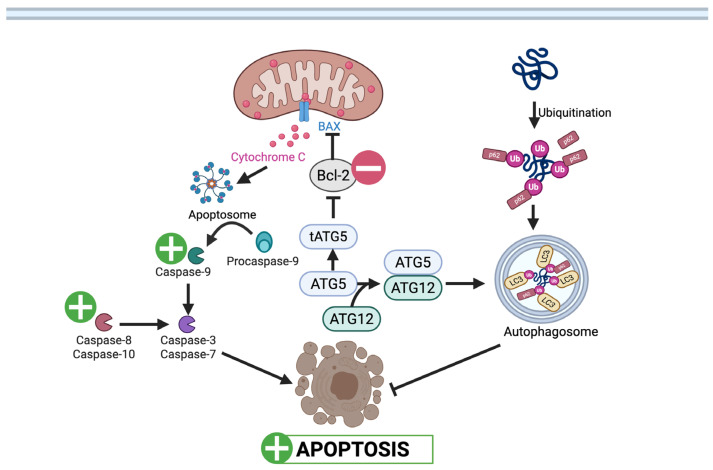
UA mediated modulation of apoptosis in CRC cells. UA inhibits the anti-apoptotic protein Bcl-2, promoting Bax-mediated cytochrome c release and caspase activation, leading to apoptosis. UA may also influence the ATG5–ATG12 pathway involved in autophagosome formation and autophagy regulation. 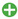: UA mediated stimulation, 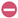: UA mediated inhibition, **┴**: inhibition. (Created in BioRender. Busselberg, D. (2025) https://BioRender.com/oatsiyd accessed on 8 November 2025).

UA may trigger apoptosis if prolonged cell cycle arrest occurs [29]. Both the intrinsic and extrinsic pathways are modulated by UA, as evidenced by the activation of initiator caspases 8 and 9, respectively (Figure 2) [16,28,30]. The anti-apoptotic pathway is affected by UA, as it alters mitochondrial membrane potential [30]. This results in the release of pro-apoptotic factors from the mitochondria, such as cytochrome c, which activate the intrinsic pathway by activating effector caspase-3 [31]. Caspase 3 then cleaves PARP, thereby promoting apoptosis by interfering with DNA repair [30,32]. UA also suppresses Bcl-2, an anti-apoptotic protein, in CRC cells (Figure 1) [33].

Additionally, UA-mediated inhibition of AKT allows FOXO transcription factors to translocate to the nucleus and upregulate pro-apoptotic genes [34]. Notably, in CD8+ memory T cells, FOXO1 activation supports T-cell maintenance and enhances anti-tumor immune surveillance (Figure 1b) [35].

#### 3.1.2. Autophagy and Mitophagy

Zhao et al. (2018) demonstrated UA induced autophagy (without apoptosis) in SW620 CRC cells at submicromolar concentrations. The suppression of this process diminished the anti-metastatic effects of UA, suggesting that autophagy is integral to its mechanism of action at lower concentrations [36]. However, this contradicts studies indicating that, in CRC, inhibition of autophagy via Atg5 knockout enhances cancer cell sensitivity to apoptosis [37]. This suggests that UA-induced autophagy may be context-dependent—potentially supportive at low doses but possibly limiting apoptosis at higher concentrations (Figure 2).

Denk et al. (2022) demonstrated that UA promotes Wnt-dependent TSCM formation by inducing mitophagy, thereby releasing PGAM5 into the cytoplasm. PGAM5 is essential for upregulating TCF1 and CD95 and supporting T cell function. This pathway triggers mitochondrial biogenesis, enhancing T cell fitness in the tumor microenvironment (Figure 1b) [38].

#### 3.1.3. Wnt/β-Catenin Suppression

Over 90% of CRCs harbor mutations that lead to overactivation of the Wnt signaling pathway [39]. Ghosh et al. (2022) demonstrated that UA can suppress this pathway in cancer cells resistant to Fluorouracil (FU) [21]. β-catenin downregulation reduces expression of proliferation and metastasis-associated genes, including SNAIL, MMP-7, cyclin D1, and c-MYC [33,40,41]. Thus, UA disrupts a central driver of CRC progression. In contrast, in CD8+ T cells, UA activates Wnt/β-catenin signaling to enhance anti-tumor immune function [38], illustrating context-dependent pathway effects.

#### 3.1.4. Chemoresistance

A significant challenge in CRC treatment is the acquired resistance to standard chemotherapeutic agents such as 5-fluorouracil (5-FU). UA has demonstrated considerable potential to overcome this resistance and restore the efficacy of conventional drugs.

Multiple studies have shown that UA can sensitize CRC cells to 5-FU. In combination treatments, UA significantly lowers the IC_50_ of 5-FU, meaning a lower drug dose is required to achieve a therapeutic effect. Additionally, 5-FU alone primarily causes arrest in the S phase while co-treatment with UA adds another block at the G2/M phase too [42]. Chemoresistant cancer cells often overexpress ATP-binding cassette (ABC) transporters, such as MDR1, BCRP, and MRPs, which actively pump chemotherapy drugs out of the cell [20]. UA has been shown to downregulate the expression of these transporters in 5-FU-resistant CRC cells, thereby increasing intracellular drug accumulation and restoring cell death. This effect is mediated by the activation of FOXO3 and the inhibition of FOXM1, a key chemoresistance-associated oncogene (Figure 1a) [21]. In a study by Núñez-Sánchez et al. (2016), physiologically relevant urolithin mixtures, especially those enriched in UA (MPhA: ~85% UA), were shown to inhibit the growth of CSC-enriched colon spheres in Caco-2 cells and primary tumor-derived cells from a patient with CRC. In addition, the percentage of ALDH-high CSCs, a chemoresistant subpopulation, was significantly reduced by UA-rich mixtures [43].

#### 3.1.5. Critical Appraisal and Translational Relevance of Current Evidence

While many studies provide detailed mechanistic insights, the majority rely on in vitro systems and often employ UA concentrations (10–100 μM) that exceed physiologically achievable levels in humans, where circulating free UA is typically low due to extensive phase-II conjugation (Table 1). In vivo data remain limited to xenograft and chemically induced models. Furthermore, sample sizes in animal studies are generally small, and few studies stratify outcomes by urolithin metabotype, a key determinant of endogenous UA exposure. These constraints highlight translational gaps that must be considered when interpreting biological effects.

### 3.2. Urolithin A and Type 2 Diabetes Mellitus

#### 3.2.1. Apoptosis and Cellular Stress vs. Autophagy

**Figure 3:**
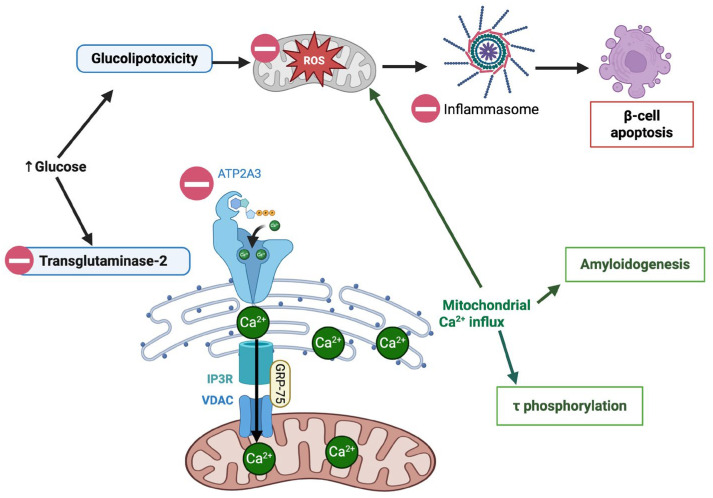
UA’s role in modulating mitochondrial function in a high (↑) glucose environment. UA reduces mitochondrial dysfunction by lowering ROS levels, preventing inflammasome activation, and β-cell apoptosis. UA also limits mitochondrial Ca^2+^ influx by inhibiting TGM-2, thereby reducing tau phosphorylation and amyloid formation. 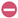: UA mediated inhibition (Created in BioRender. Busselberg, D. (2025) https://BioRender.com/x9iuf4l accessed on 8 November 2025).

Across all cell types, UA exhibits strong protective effects against cellular stress and apoptosis (Table 2). By blocking the action of Transglutaminase 2 (TGM2)—an enzyme that promotes the formation of contact sites between the ER and mitochondria, known as Mitochondria-associated membranes (MAMs)—UA lowers the excessive calcium influx from the endoplasmic reticulum (ER) into the mitochondria and avoids subsequent mitochondrial calcium overload. This regulation helps to reduce neuronal stress by reducing amyloid formation and Tau hyperphosphorylation, which are markers of neurodegenerative processes (Figure 3) [44,45]. Consistent with these findings, Xiao et al. (2023) demonstrated that UA alleviates cognitive impairment in T2DM mice by reducing Tau hyperphosphorylation, ER stress, and oxidative damage in the brain. In vitro, UA protected hippocampal neurons and HT22 cells from high-glucose and oxidative stress-induced apoptosis by downregulating Atp2a3, a regulator of calcium homeostasis and ER stress [46].

A primary mechanism by which UA exerts its beneficial effects, particularly in diabetic contexts, is by enhancing cellular quality-control processes, such as the targeted mitochondrial cleanup process, mitophagy. In T2DM, persistent metabolic stress and high glucose levels impair autophagy, leading to the accumulation of damaged cellular components and ultimately triggering cell death [47]. UA counters this by reactivating autophagic flux and stabilizing mitochondrial function.

The anti-apoptotic and protective effects of UA are observed across numerous cell types, often directly linked to the activation of autophagy and mitophagy (Table 2). In pancreatic β-cells of diabetic mice, UA decreased cleaved caspases, indicating a significant reduction in apoptosis. In vitro, UA protected MIN6 pancreatic cells from glucolipotoxicity-induced apoptosis by restoring mitochondrial membrane potential and activating autophagy [48].

**Figure 4:**
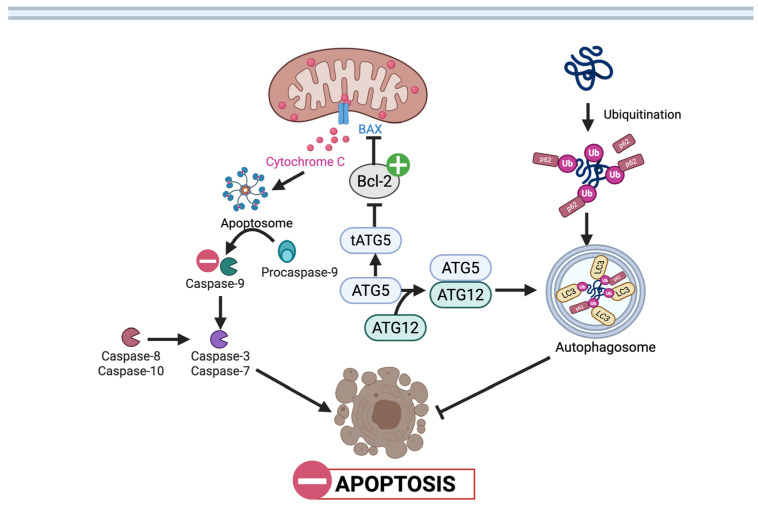
Urolithin-A mediated modulation of apoptosis in pancreatic β cells. UA stabilizes Bcl-2 to prevent cytochrome c release and caspase activation, thereby reducing apoptosis. UA also enhances autophagic clearance of damaged proteins through ATG5–ATG12 and p62 signaling. 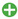: UA mediated stimulation, 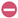: UA mediated inhibition, **┴**: inhibition. (Created in BioRender. Busselberg, D. (2025) https://BioRender.com/0v18m6c 8 November 2025).

**Figure 5:**
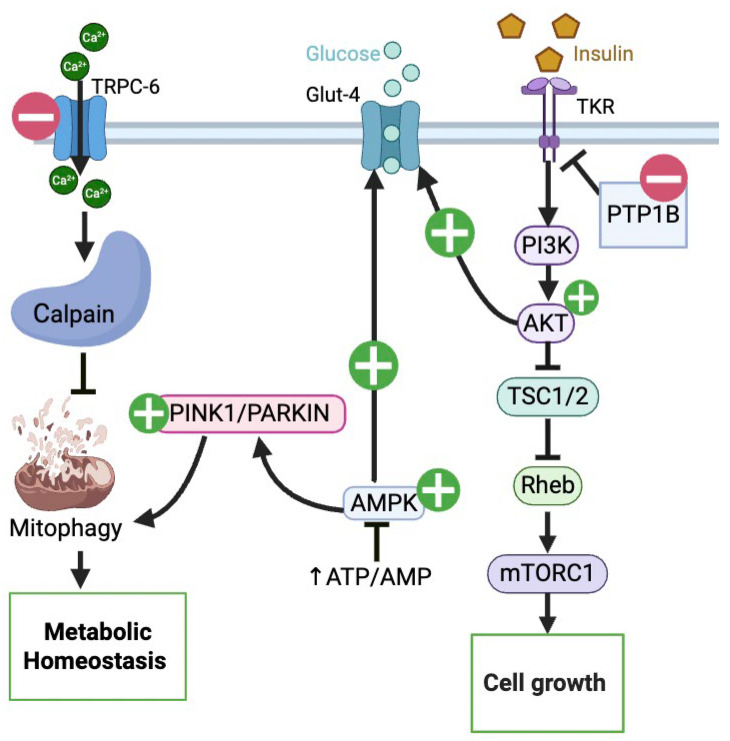
Pathways modulated by UA in T2DM: UA promotes mitophagy by modulating TRPC6–calpain signaling and activating the PINK1/Parkin pathway, supporting mitochondrial quality control. UA-induced activation of the AMPK and PI3K/AKT/mTOR pathways enhances glucose uptake via GLUT4, improves cell growth, and reduces PTP1B-mediated insulin resistance. 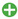: UA mediated stimulation, 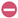: UA mediated inhibition, **┴**: inhibition. (Created in BioRender. Busselberg, D. (2025) https://BioRender.com/3w2bhrn 8 November 2025).

These protective effects are directly mediated by its ability to activate autophagy via the AKT/mTOR signaling pathway, as confirmed in studies showing that blocking autophagy with inhibitors, such as chloroquine, eliminated the anti-apoptotic benefits of UA [49]. In podocytes exposed to high glucose, a model for diabetic kidney disease, UA improved cell survival and decreased apoptosis through the Bcl-2/caspase-3 pathway (Figure 4) and by increasing autophagy markers [50]. Similarly, UA has been shown to protect against diabetic kidney disease and hypoglycemia-induced heart injury by activating the PINK1/Parkin mitophagy pathway [51,52]. In the skeletal muscle of mice with diet-induced insulin resistance, UA has been shown to increase PINK1/PRKN (Parkin) mitophagy pathways (Figure 5) [53]. Paradoxically, in vascular smooth muscle cells, UA suppresses AKT phosphorylation, leading to downregulation of genes such as c-Myc and Cyclin D1, thereby preventing vascular smooth muscle proliferation [54,55]. In a study by Fang et al. (2025), UA demonstrated the ability to revitalize endothelial progenitor cells by activating Parkin-mediated mitophagy (Figure 5) [56]. Likewise, a study by Tang et al. (2023) found that UA alleviated T2DM-associated cognitive dysfunction in T2DM mice by restoring mitophagy in hippocampal neurons [57].

**Table 2:** Major studies exploring effects of UA in T2DM and Associated Complications.

System/ Complication	Model/System	UA Dose/Exposure	Mechanism(s)	Key Findings	References
Pancreatic β-cell survival	Diabetic mice; MIN6 β-cells	50 mg/kg/day; 11.5 μg/mL	↑ Autophagy (LC3II ↑, SQSTM1/p62 ↓); ↓ Caspase-3/1; ΔΨm restoration	↓ β-cell apoptosis; ↑ β-cell viability and morphology	[48]
Obesity/insulin resistance	HFD & ob/ob mice; Adipocytes	30 mg/kg/day; 20 μM	↑ BAT thermogenesis; ↑ iWAT browning; ↑ UCP1/PGC-1α; ↑ Dio2 → ↑ T3	↓ Adiposity; ↑ Insulin sensitivity	[23]
Insulin resistance (liver and skeletal muscle)	DBA/2J mice; Hepatocytes	0.1% dietary UA; 10 μM	↑ Mitophagy (PINK1/PRKN); ↑ MFN2; ↑ mtDNA; ↓ Proton leak	↓ Fasting glucose; ↑ Adiponectin; Improved IPTT/GTT	[53]
Diabetic retinopathy	STZ rats; HRECs	2.5 mg/kg/day; 10 μM	↑ Nrf2/HO-1; ↓ IL-6, IL-1β, TNF-α; ↓ MDA; ↑ SOD/GSH	↓ Oxidative stress; ↓ VEGF-mediated leakage; Improved retina	[58]
Cognitive dysfunction	T2DM mice; HT22 neurons	200 mg/kg/day; 5 μM	↓ ER stress (ATF6, CHOP); ↓ Atp2a3; ↓ Tau phosphorylation	↑ Cognition; ↓ Apoptosis	[46]
Gut barrier dysfunction	T2DM mice; Caco-2 cells	200 mg/kg; 10 μM	↑ Tight junctions (ZO-1, Occludin, Claudin 1); ↓ TLR4/Myd88; ↓ NLRP3; ↑ *N*-glycosylation genes	↑ Gut integrity; ↓ Inflammation; ↑ Cognition	[59]
Podocytopathy (kidney)	Mouse podocytes (HG)	10 μM (beneficial); (cytotoxic at 100 μM)	↓ ROS; ↑ Bcl-2; ↑ Autophagy (LC3B, ATG5); ↑ Nephrin	↑ Podocyte survival	[50]
DKD— tubular injury	STZ mice; HK-2 cells	50 mg/kg/day; 20 μM	↑ Mitophagy ↓ TRPC6–calpain-1; ↓ Cytokines	↓ Tubular fibrosis & inflammation	[51]
Wound healing (angiogenesis)	STZ rats; EPCs	25 mg/kg/day; 20 μM	↑ Mitophagy (Parkin); ↓ ROS ↑ EPC proliferation	↑ Wound closure; ↑ Neovascularization	[56]
Vascular dysfunction (HG)	STZ rats; VSMCs	Juice → UA; 5–40 μM	↓ AKT phosphorylation ↓ β-catenin (c-Myc, cyclin D1)	↑ Endothelial function ↓ VSMC proliferation	[54]
Diabetic cardiomyopathy	STZ rats (T1D model)	2.5 mg/kg/day	↑ SIRT1 → deacetylation of Nrf2, FOXO1, NF-κB; ↑ MnSOD, GSH; ↓ ROS, TNF-α, IL-6	↑ Cardiac function; ↓ Fibrosis (TGF-β1, Smad3, Col1A1); ↓ Apoptosis; Effects abolished by SIRT1 inhibitor	[60]

**↑** indicates increase, **↓ **indicates decrease.

#### 3.2.2. Glucose Metabolism and Insulin Signaling

Modulating glucose metabolism and insulin signaling is another way UA exerts its anti-diabetic effects. Skeletal muscle, the primary site for glucose disposal, appears to be a main target. In rat skeletal muscle cells (L6 myotubes), UA increased glucose uptake in a dose-dependent manner, even in the absence of insulin [22]. Mechanistically, this occurs through the simultaneous activation of the PI3K/AKT and AMPK signaling pathways. Activation of these pathways promotes the movement of GLUT4 transporters to the plasma membrane, boosting glucose entry into the cell (Figure 3). These cellular effects result in tangible benefits in vivo. In diabetic KK-Ay/Ta mice, oral UA significantly lowered glucose levels at 90 and 120 min after glucose injection [22]. UA also influences glucose metabolism by targeting key enzymes involved in carbohydrate metabolism and insulin signaling. In silico and in vitro analyses have demonstrated that UA can bind to and inhibit α-amylase, α-glucosidase, and lipase, and thereby lower postprandial hyperglycemia [61,62]. UA-mediated inhibition of DPP-4 prolongs GLP-1 activity and enhances insulin secretion [62]. UA also inhibits aldose reductase [57], a key enzyme that contributes to diabetic complications by converting high glucose levels into sorbitol. This process produces ROS that may contribute to several diabetes-related issues, including nephropathy, cardiovascular disease, and neuropathy [63]. A randomized controlled trial by Barone Lumaga et al. (2024) reported a significant reduction in serum DPP-4 activity and lower fasting blood glucose levels among participants who consumed foods that increased their UA levels [64].

In silico analyses suggest that UA may inhibit protein tyrosine phosphatase 1B (PTP1B), a negative regulator of insulin receptor signaling (Figure 3) [65]. Computational docking studies further indicate that UA could interact with the insulin receptor (IR) and retinol-binding protein 4 (RBP4), suggesting a potential role in enhancing insulin receptor signaling and mitigating RBP4-associated insulin resistance [66].

#### 3.2.3. Anti-Inflammatory and Antioxidant Effects

UA exhibits notable anti-inflammatory and antioxidant effects. In humans with T2DM, consuming red raspberries, which leads to UA production, significantly reduces the inflammatory marker hsCRP [67]. UA also prevents the formation of Advanced Glycation end products (AGEs), which are linked to diabetic complications due to increased production of ROS on AGE binding to its receptor (RAGE). It accomplishes this by scavenging reactive carbonyls like methylglyoxal and modifying protein structures to inhibit glycation [68]. In preclinical models, UA has been shown to lower inflammatory cytokines such as IL-6, TNF-α, and MCP-1 in diabetic kidney disease [51]. UA also demonstrated the ability to improve the integrity of tight junctions, thereby helping to inhibit inflammation caused by leaked LPS [59]. Additionally, in models of diabetic retinopathy, UA reduced inflammation (IL-6, IL-1β, TNF-α) and oxidative stress by increasing levels of SOD and GSH while decreasing MDA, effects associated with activation of the Nrf2/HO-1 pathway [58]. Consistently, studies show that UA suppresses pro-inflammatory cytokines such as IL-1β and TNF-α by downregulating NF-κB signaling in adipose tissue [68]. Additionally, UA promoted a phenotypic switch from pro-inflammatory M1 macrophages to anti-inflammatory M2 macrophages, reducing immune cell infiltration into adipose tissue [69,70].

#### 3.2.4. Modulating Adipose Tissue and Systemic Health

UA shows significant potential in modulating adipose tissue and enhancing systemic health. Toney et al. (2019) demonstrated that UA supplementation improves systemic insulin sensitivity in high-fat diet-fed mice, reduces hepatic triglyceride accumulation, and promotes the upregulation of mitochondrial β-oxidation-related genes, such as Cpt1 and Sirt1 [69]. Other studies have shown that UA mediates the downregulation of adipogenesis-related genes, such as PPAR-γ, FABP4, and adiponectin [62]. In adipose tissue, UA attenuated adipocyte hypertrophy [62,69]. UA has been found to reduce both diet-induced and genetic obesity, which in turn improves overall glucose uptake and lowers endogenous hepatic glucose production. A key mechanism behind this is its ability to promote thermogenesis in brown adipose tissue (BAT) and the “browning” of white adipose tissue (IWAT). This effect occurs by increasing the local production of the active thyroid hormone T3 (triiodothyronine) within adipose tissue, which then triggers the expression of thermogenic genes, particularly UCP-1 and PGC-1α [23]. Together with the mechanistic evidence that UA reduces adiposity, enhances insulin sensitivity, and mitigates systemic inflammation, a cross-sectional study of Spanish adolescents reported that higher urinary microbial phenolic metabolites (including urolithin-related compounds) were inversely associated with metabolic-syndrome features. This inverse link could suggest a possible protective, early-life role for endogenous UA against the trajectory toward insulin resistance and T2DM [71].

### 3.3. Shared Mechanisms of UA’s Role in CRC and T2DM

UA’s capacity to modulate cellular stress responses via overlapping signaling pathways is a key component of its role in T2DM and CRC. Chronic inflammation, elevated oxidative stress, and mitochondrial dysfunction are the leading causes of both diseases. In T2DM, persistent hyperglycemia and lipotoxicity destabilize mitochondrial quality control, amplifying ROS production and ultimately leading to β-cell apoptosis and tissue damage [72]. Excess ROS also encourages additional DNA damage in CRC cells [73]. By stimulating the Nrf2 axis and increasing antioxidant enzymes, such as HO-1, UA reduces the burden of ROS in most tissues [68]. An important exception is the UA-mediated enhancement of ATRA-induced O_2_^−^ production in macrophages, which promotes the phagocytosis of cancer cells [74]. In addition to fostering cardiomyocyte and podocyte survival in diabetic conditions (Table 1), this anti-oxidation action also inhibits the progression of cancer in CRC patients [35]

The regulation of mitophagy through the PINK1/Parkin pathway represents a second significant intersection. Insulin resistance and cell apoptosis are exacerbated in T2DM due to impaired mitophagy, leading to the accumulation of dysfunctional mitochondria in β-cells, podocytes, and cardiomyocytes. By encouraging the selective removal of damaged organelles, UA restores mitochondrial fidelity [48,51,52], Meanwhile, in CRC, the same pathway contributes to immunity: In CD8 T cells, UA-induced mitophagy improves depleted T cell populations, memory stem cell subsets, and overall anti-tumor immunity [39]. This highlights the context-dependent duality of UA by turning what is a cytoprotective mechanism in metabolically stressed tissues into an immunomodulatory lever in the tumor microenvironment.

Moreover, UA can regulate both PI3K/AKT/mTOR and β-catenin signalling pathways, which are disrupted in both CRC and T2DM. However, UA exhibits distinct therapeutic aims in the two conditions. UA inhibits AKT phosphorylation in CRC cells (Figure 1) while upregulating this growth-promoting pathway in T2DM (Figure 5). This reciprocal regulation demonstrates that UA can specifically alter pathways depending on the cellular environment, thereby limiting the growth of cancer cells and enhancing cell survival when metabolic homeostasis is disrupted.

Apoptosis is another typical mechanism modulated by UA, highlighting its ability to modulate the same pathway differently across environments. To guarantee tumor cell removal in CRC, UA induces apoptosis through intrinsic and extrinsic caspase cascades [56], On the other hand, UA prevents β-cells and podocytes from dying by preserving ΔΨm, thereby stabilizing mitochondrial membranes and reducing caspase activation [49,51]. Finally, in the context of inflammation, in diabetic tissues, UA suppresses IL-6, TNF-α, and MCP-1 while dampening TLR4/Myd88 and NLRP3 inflammasome activity, thereby curbing tissue injury [67,69]. In CRC, the same pathways are implicated in tumor-promoting inflammation, and UA’s dampening effect restricts cytokine-driven proliferation and metastasis [28].

The combination of these functions makes UA a molecular “balancer,” reducing the diabetic microenvironment that increases the risk of CRC while also directly preventing tumor growth (Table 3). In addition to explaining UA’s potential for managing comorbidities, these pleiotropic effects highlight the therapeutic complexity of a molecule that can reprogram cellular fate in response to context.

## 4. Discussion

UA’s anti-cancer actions are strongly supported by their positive effects on T2DM, which is a significant risk factor for CRC [77,78]. T2DM promotes colorectal tumor growth through mechanisms such as hyperinsulinemia, which activates the pro-growth PI3K/AKT/mTOR signaling pathway in colonic epithelial cells [79]. Mechanistically, UA intake by a diabetic patient downregulates the AKT/β-catenin pathway in colonic cells [21], thereby reducing the otherwise increased risk of developing CRC. Furthermore, UA reduces inflammation and activates the Nrf2 antioxidant pathway, which helps combat oxidative stress found in both T2DM and CRC (Table 3). Oxidative-stress modulation by UA is primarily protective (e.g., Nrf2/HO-1 activation in diabetic tissues and decreasing ROS levels in CRC cells), but context-specific pro-oxidant nudges may be therapeutically beneficial. For example, UA can amplify ATRA-stimulated superoxide production in macrophages, a cytotoxic burst that could conceivably aid tumor cell clearance [74]. This duality suggests the need for nuance: in patients with fragile tissues (e.g., advanced nephropathy), it is advisable to avoid combining UA with agents that exacerbate the respiratory burst [80]. Moreover, UA sensitizes CRC to standard therapies: it downregulates chemoresistance mechanisms (MDR1, BCRP, MRP transporters) via the FOXO3–FOXM1 pathway, thereby restoring 5-FU efficacy [21]. This chemosensitization is a double benefit in diabetic CRC, where patients often have worse chemo-response [81]. By targeting CRC cells while also disrupting the high-glucose, pro-inflammatory environment that fuels cancer, UA offers combined therapeutic potential for patients facing both conditions.

A significant property of UA that warrants further exploration for determining its therapeutic potential in various diseases is its pathway polarity. UA tends to favor glucose uptake via AMPK and PI3K/AKT signaling. Consistent with this, UA improves skeletal-muscle glucose handling and GLUT4 trafficking in preclinical models [22]. The clinical implication is that synergy with metabolic treatments that also act through the AMPK pathway can be expected, such as metformin [82]. In contrast, UA can inhibit downstream β-catenin outputs and dampen AKT activity in endothelial and CRC cells, thereby lowering pro-growth signaling [21,55]. This bidirectionality is not contradictory; it appears to be context-dependent and advantageous: UA can enhance insulin action in muscle tissues, while simultaneously suppressing oncogenic AKT/β-catenin activity in the colon and its vasculature. However, it is essential to monitor for UA’s pharmacodynamic interactions with oncologic agents that inhibit PI3K/AKT, as these agents, when given together, could lead to side effects such as hyperglycemia. Beyond muscle, UA drives thermogenic programming and browning of adipose tissue, a systemic lever for improving insulin sensitivity [23]

Despite its promises, a major translational limitation is the lack of human clinical trials evaluating UA’s efficacy in CRC or T2DM. Although human trials remain limited, several dietary and supplementation studies have clarified the translational landscape of UA metabolism, exposure, and safety. In healthy adults, Mosele et al. (2015) showed that four weeks of pomegranate-juice intake increased fecal UA and phenolic catabolites, yet only ~67% of participants were UA producers, underscoring large inter-individual variability in gut biotransformation [83]. In CRC patients, Núñez-Sánchez et al. (2014) confirmed tissue delivery of UA and Isourolithin A after 15 days of pomegranate-extract supplementation (900 mg/day), detecting up to ~7 µM UA in normal colon mucosa but markedly lower levels in tumor tissue, suggesting local metabolism and conjugation differences [84]. From a metabolic perspective, Zhang et al. (2020) reported that four weeks of red-raspberry consumption enhanced circulating microbial metabolites, including urolithins, in prediabetic subjects [85], while Barone Lumaga et al. (2024) demonstrated that a two-week high-fiber, walnut-enriched breakfast increased urinary UA-sulfate and modestly reduced fasting glucose and DPP-IV activity [64]. Collectively, these trials indicate that dietary ellagitannins can reproducibly enhance UA generation and yield mild improvements in metabolic biomarkers, but exposure remains modest and highly variable.

Quantitatively, human pharmacokinetic studies show that even after a single 500 mg oral UA dose (Mitopure), plasma UA-glucuronide peaks at approximately 480 ng/mL (~2 µM) within 6 h [86]. Hence, we can infer that systemic levels of UA aglycones would be much less than even 2 µM, due to extensive phase II metabolism. Colon-tissue concentrations after repeated pomegranate-extract dosing also lie in the low-µM range (~7 µM) [84], consistent with conjugated rather than free forms dominating systemic circulation. These levels are one to two orders of magnitude below the 10–100 µM concentrations commonly used in vitro to elicit cytotoxic, anti-inflammatory, or insulin-sensitizing effects in cell models (Table 1 and Table 2). Consequently, many mechanistic claims regarding direct UA cytotoxicity or insulin-signaling modulation remain speculative, as such exposures are unlikely to occur in vivo. Verified human outcomes are presently limited to measurable increases in conjugated UA metabolites, modest improvements in glycemic or inflammatory markers, and consistent reports of tolerability at doses up to 1 g/day over several months [87,88]. While extensive preclinical data demonstrate UA’s anticancer and metabolic benefits, these findings have yet to be validated in controlled human studies. Several studies have used UA concentrations as high as 100 μM to demonstrate the cytotoxic and anti-proliferative effects of UA on CRC cell lines in vitro [25,28]. However, at these concentrations, UA may also exert cytotoxic effects on podocytes and hepatocytes under high-glucose conditions (Table 2).

Exposure biology further complicates translation. UA production depends on microbiome “metabotypes,” with only a subset of individuals (metabotype A) efficiently converting ellagitannins to UA [89]. This capacity is reduced in insulin resistance and CRC, leading to lower circulating UA-derived metabolites [83,84]. Local glucuronidation also constrains free UA signaling; for example, UA metabolism proceeds more slowly under high-glucose conditions, potentially enhancing local protective signaling [50]. However, preclinical CRC studies show that physiologically relevant UA exposures can still modulate metabolic fitness and chemosensitivity [18,21,42].

Two pragmatic routes follow. (1) Low-dose, pathway-modulating strategy: use UA as chemopreventive or as an adjunct to standard therapies (5-FU, immunotherapy), aiming to improve mitophagy, barrier integrity, and T-cell fitness rather than to achieve direct tumor cytotoxicity. (2) High-local-dose, colon-targeted delivery: deploy pH-responsive or microbiota-activated formulations to release free UA at the tumor bed while keeping systemic levels within the protective range [90]. Given UA’s transporter-modulating and immune-conditioning properties, rational combinations should prioritize 5-FU–based regimens and T-cell–directed immunotherapies with pharmacodynamic readouts (e.g., mitophagy signatures, effector-memory phenotypes) captured alongside glycemic indices [21,24,42].

Even with optimized delivery, patient context matters. UA’s T-cell benefits are attractive for oncology, especially considering UA’s ability to re-prime CD8^+^ T cells towards memory-stem phenotypes, restoring depleted pools and enhancing persistence [38]. Practically speaking, this implies that UA may have a very significant effect on T2DM with CRC when administered in conjunction with immunotherapies, by strengthening the T-cell response. Still, in autoimmunity-prone settings (e.g., type 1 diabetes), any intervention that expands long-lived memory pools warrants caution and close monitoring due to the potential risk of promoting diseases like type 1 diabetes [91]. Vascular biology is another key consideration: short-term endothelial AKT inhibition may be anti-angiogenic and thus desirable for tumors [55] yet could be counterproductive in patients requiring collateral formation (e.g., limb ischemia). Prospectively stratifying by insulin-resistance status and urolithin metabotype, while capturing UA conjugates and free aglycone in plasma/stool, will be essential to connect exposure with antitumor and metabolic pharmacodynamics [24].

Implications for Future Research and Use:

- Targeted delivery strategies: Advance UA formulations (e.g., pH-responsive polymers, nanoparticles) to concentrate high doses in colonic tumors while limiting systemic exposure. Nanocarriers designed to release UA in the tumor microenvironment could widen its therapeutic window [92].
- Dose and regimen optimization: Map the dose–response continuum of UA’s effects. Careful titration is needed to separate its anti-CRC (high-dose) and anti-diabetic (low-dose) actions. For example, intermittent high-dose pulses might kill tumor cells while daily low-dose maintains metabolic control.
- Combination therapy trials: Given UA’s ability to sensitize CRC to chemotherapy and to enhance antitumor T-cell responses, testing UA alongside standard chemo/immunotherapies is warranted. Early-phase clinical trials could assess the effects of UA supplementation in CRC patients with metabolic syndrome, monitoring tumor markers, glycemic indices, and immune phenotypes.
- Patient stratification and biomarkers: Identify biomarkers (e.g., gut metabotype, insulin resistance status) to select patients most likely to benefit from UA. Only some individuals efficiently convert ellagitannins to UA.
- Safety assessments: Evaluate UA’s effects in autoimmunity and healthy tissues. Its immune-boosting properties should be tested for safety in autoimmune disease models to ensure it does not exacerbate β-cell autoimmunity. Long-term toxicology should confirm that sustained UA levels (even via targeted delivery) are well tolerated.

## 5. Conclusions

Urolithin A, a gut microbiota–derived metabolite of dietary ellagitannins, emerges as a molecular bridge between CRC and T2DM. Both diseases share dysregulated insulin/IGF-1 signaling, chronic inflammation, oxidative stress, and mitochondrial dysfunction—mechanistic axes that UA can modulate through AMPK activation, PI3K/AKT/mTOR inhibition, and restoration of mitophagy and autophagy. This convergence positions UA as a dual-target compound that can simultaneously influence metabolic and oncogenic pathways.

Translationally, preclinical evidence supports UA’s antitumor and insulin-sensitizing effects. Still, human data remain limited to small trials demonstrating increased systemic UA exposure, modest glycemic improvements, and tissue accumulation without definitive clinical outcomes. Inter-individual differences in UA production (“metabotypes”), rapid conjugation, and uncertain effective doses represent major barriers to therapeutic application.

Future research should prioritize biomarker-guided, metabotype-stratified clinical trials that define optimal UA exposure, establish pharmacokinetic–pharmacodynamic relationships, and test colon-targeted or systemic delivery strategies. Integrating microbiome engineering with UA supplementation may enhance translational efficacy. Overall, UA exemplifies how microbial metabolites can reframe chronic disease prevention—linking diet, microbiota, and host signaling in the shared pathogenesis of CRC and T2DM.

## Figures and Tables

**Table 1:** Comparative Analysis of UA Studies in CRC.

Study	Model	UA Dose	Main Findings (↑ */↓ **)	Advantages	Limitations
González-Sarrías et al., 2015 [42]	Caco-2, SW480, HT-29 (human CRC)	10 µM, 20 µM (with 5-FU)	**↓** IC_50_ of 5-FU/5′DFUR; chemosensitization	Multiple colon cancer cell lines; Use of concentrations achievable in the colorectum	No in vivo validation; limited to combo treatment (UA + 5-FU)
Zhao et al., 2018 [36]	SW620 (human CRC)	0.05–15 µM	At sub-µM: **↑** Autophagy	Shows activity at sub-micromolar doses—suggests plausible colonic relevancy	Single CRC line only; no animal/human confirmation
Norden & Heiss (2019) [18]	HCT116 (human CRC) WT vs. p53^−^/^−^	IC_50_ ≈ 19 µM (72 h)	**↑** p53, **↑** p21; **↓** glycolysis (via p53/TIGAR)	Mechanistic insight into p53 dependency—potential biomarker for response	Effects depend on tumor p53 status; no in vivo or clinical data
El-Wetidy et al., 2021 [16]	HT-29, SW480, SW620 (human CRC)	IC_50_: ~25.5 µM HT-29; 38.1 µM SW480; 53.6 µM SW620 (at 48 h)	G2/M arrest; **↑** p53 & p21; **↓** Bcl-2; **↑ **cytochrome-c release; **↑ **caspase-3 activation; **↑** Reactive Oxygen Species (ROS)	Multiple cell lines tested.	No in vivo experiments; physiological relevance questionable
Ghosh et al., 2022 [21]	SW480, HCT116 (5-FU resistant lines); NRG mice xenografts; AOM/DSS mice	In vitro: 10–50 µM; In vivo: UroA 20–40 mg/kg PO (mice); 5-FU 20 mg/kg i.p	UA ± 5-FU: **↓** Viability, **↓** Invasion, **↓ **EMT markers, **↓ **drug transporters (MDR1, BCRP, MRP2/7); **↓** tumor growth in xenograft & AOM/DSS models	in vitro and in vivo model tested; immunocompetent and immunodeficient in vivo mouse models tested.	UAS03 is synthetic (not naturally produced by gut microbiota), so human applicability depends on safety/PK in humans. No direct patient data or clinical validation
González-Sarrías et al., 2009 [33]	Caco-2 (human) + rat colon (in situ buffer experiments)	40 µM *n* = 3	**↑** Phase I/Phase II detox enzymes (**↑ **CYP1A1, **↑** UGT1A10); inhibited sulfotransferases (favoring glucuronidation)	It compared in vitro (Caco-2), in situ (rat colon perfusion), and in vivo (rat feeding) models.	Inability to reproduce the in vitro findings in the in vivo animal colon, suggesting the in vitro model (compounds in buffer) lacks physiological relevance for this specific endpoint
González-Sarrías et al., 2014 [25]	Caco-2, SW480, HT-29	100 µM (aglycones & glucuronides tested)	S and G2/M arrest Uro-A most active. Glucuronidation: ↓ activity	Used multiple cell lines; it directly tested and compared the biological activity of urolithin aglycones against their glucuronide metabolites.	High concentrations; No in vivo testing
González-Sarrías et al., 2017 [26]	Caco-2 and CCD18-Co (normal colonic cells)	IC_50_ ≈50 µM Uro-A, ~70 µM IsoUro-A)	**↓** Proliferation, S/G_2_ arrest, **↑** Apoptosis at ~50–70 µM; conjugated forms inactive; higher glucuronidation in IsoUro-A	Compares isomeric forms and conjugation rates.	High concentrations; physiologic relevance uncertain; lack of in vivo validation
Giménez-Bastida et al., 2020 [19]	HCT116 (p53WT), Caco-2, HT-29	10 µM chronic exposure (up to >2 weeks)	**↑** cellular senescence (**↑** p53/p21) and anti-clonogenic effects	Focus on long-term low-dose exposure (physiologically relevant colonic concentrations)—strong translational logic	No animal data; emphasis on senescence rather than cell death
Núñez-Sánchez et al., 2016 [43]	Caco-2 colonospheres; primary patient-derived CRC CSCs	Mixtures: C1 = 0.85 mM; C2 = 17 mM (UA ≈ 85% of mix)	**↓ **colonosphere **↓ **ALDH^+^	Uses patient-derived CSCs (more clinically relevant)	Concentrations very high; unrealistic physiologic exposure; mixture study obscures single-compound effect; no in vivo data
Tortora et al., 2018 [27]	Pirc rats (APC^+/−^ model) + CRC cell lines; ex vivo tissue	Diet-derived UA (pomegranate mesocarp)—in vitro: UA 25 µM + 2.5 mM sodium butyrate	**↓ **precancerous lesions (MDF); **↑** apoptosis in lesions	Combines in vitro & in vivo evidence	UA effect shown only with co-treatment (butyrate/diet matrix); small *n* (rats: *n* = 4); Pirc model = FAP representation (not general CRC)
Tiwari et al., 2024 [28]	HT-29 (human colon adenocarcinoma)	IC_50_ ≈120 µM	**↓** Viability (IC_50_ ≈ 120 µM); **↑** p53/p21, **↑** caspase activation; **↓** Bcl-2, AKT1/2	Detailed pathway predictions and in vitro validation	IC_50_ extremely high (120 µM)—far above realistic plasma levels; single cell line; no in vivo data

* **↑ **indicates increase, ** **↓** indicates decrease.

**Table 3:** Pathways affected by UA that is common to its effect in CRC and T2DM.

Scheme Molecule/Pathway	Effect in CRC	Effect in T2DM	References for CRC	References for T2DM
Wnt/β-catenin	In T-cells: ↑ In CRC cells: ↓	In VSMC: ↓ In β-cells: ↑	[21,38]	[22,49,54]
Apoptosis (p53, p21, caspases, Bax/Bcl-2)	In C.R.C cells: ↑	In β-cells: ↓ Podocytes: ↓	[16,18,28]	[48,50]
Autophagy & Mitophagy (PINK1/Parkin, ULK1, LC3)	In T cells: ↑	In β-cells: ↑ In Podocytes: ↑ In EPC: ↑	[36,38]	[48,51,56]
Inflammation (TLR4/Myd88/NLRP3, cytokines)	In CRC cells: ↓ IL-6, ↓ TNF- α	In Retinal cells: ↓ IL-6, ↓ IL-1β, and ↓ TNF-α In Gut/Brain cells: ↓ TLR4/Myd88, ↓ NLRP-3	[21]	[51,58,59]
Oxidative Stress (ROS, Nrf2/HO-1)	In CRC cells: ↓ R.O.S, MDA, ↑ S.O.D.	In β-cells: ↓ MDA, ↓ GSH In retinal cells: ↑ Nrf2/HO-1	[75]	[49,58]
Aryl Hydrocarbon Receptor (AhR) pathway	In colonic cells: ∴↑ gut barrier integrity	In neurons: ↑ ∴↑ neuroprotection	[76]	[45]

**↑ **indicates increase, **↓** indicates decrease, ∴ indicates therefore.

## Data Availability

No datasets were generated or analyzed during the current study.

## Abbreviations

The following abbreviations are used in this manuscript:

Aβ	Amyloid-β
ATG5	Autophagy related 5
ATRA	All-trans retinoic acid
AKT	Protein kinase B (AKT)
AMPK	AMP-activated protein kinase
BAT	Brown adipose tissue
BAX	Bcl-2 associated X protein
BCL-2	B-cell lymphoma 2 (anti-apoptotic protein)
CRC	Colorectal cancer
DPP-4	Dipeptidyl peptidase-4
DRP1	Dynamin-related protein 1 (mitochondrial fission regulator)
EPC(s)	Endothelial progenitor cell(s)
ER	Endoplasmic reticulum
GLUT4	Glucose transporter type 4
GSH	Glutathione
HO-1	Heme oxygenase-1
IR	Insulin receptor
IWAT	Inguinal (subcutaneous) white adipose tissue
LC3	Microtubule-associated protein 1 light chain 3 (autophagy marker)
MAM(s)	Mitochondria-associated membranes
MCP-1	Monocyte chemoattractant protein-1 (CCL2)
MDA	Malondialdehyde
mtROS	Mitochondrial reactive oxygen species
NLRP3	NOD-, LRR- and pyrin domain-containing protein 3 (inflammasome)
Nrf2	Nuclear factor erythroid 2-related factor 2
OCR	Oxygen consumption rate
PI3K	Phosphoinositide 3-kinase.
PINK1	PTEN-induced kinase 1
PRKN (Parkin)	Parkin RBR E3 ubiquitin protein ligase (PRKN)
PTP1B	Protein tyrosine phosphatase 1B
PSD95	Postsynaptic density protein 95
PTEN	Phosphatase and tensin homolog (if used in text)
ROS	Reactive oxygen species
SOD	Superoxide dismutase
SQSTM1/p62	Sequestosome 1 (p62; autophagy adaptor)
T2DM	Type 2 diabetes mellitus
TGM2	Transglutaminase 2
TSCM	T memory stem cell (memory-stem CD8^+^ T cell)
UCP1	Uncoupling protein 1 (thermogenesis marker)
UA	Urolithin A
ULK1	Unc-51 like autophagy activating kinase 1
ΔΨm	Mitochondrial membrane potential (delta psi m)
